# Manual therapy in the treatment of patients with hemophilia B and inhibitor

**DOI:** 10.1186/s12891-018-1934-9

**Published:** 2018-01-22

**Authors:** Rubén Cuesta-Barriuso, Roberto O. Trelles-Martínez

**Affiliations:** 1 Department of Physiotherapy, European University of Madrid, Madrid, Spain; 2 Royal Victoria Eugenia Foundation, Madrid, Spain; 3 Fishemo SLU-Spanish Federation of Hemophilia, Madrid, Spain; 4 Department of Hematology, Clínico San Carlos Hospital Madrid, Madrid, Spain

**Keywords:** Hemophilia inhibitor, Joint damage, Physiotherapy, Case report, Hemophilia arthropathy, Inhibitor, Manual therapy, Hemophilic arthropathy, Safety

## Abstract

**Background:**

The main clinical manifestations of hemophilia are muscle and joint bleeding. Recurrent bleeding leads to a degenerative process known as hemophilic arthropathy. The development of inhibitors (antibodies against FVIII/FIX concentrates) is the main complication in the treatment of hemophilia. The objective was to assess the safety and efficacy of manual therapy treatment in a patient with hemophilia and inhibitor.

**Case presentation:**

A 26-year-old patient with hemophilia B and inhibitor received physiotherapy treatment based on manual therapy for 3 months, with a frequency of 2 sessions per week. The joint status was evaluated using the *Hemophilia Joint Health Score*; pain was assessed with the *Visual Analog Scale*; and the range of movement was evaluated using a universal goniometer. The patient developed no joint bleeding in the knees or ankles as a result of the physiotherapy treatment. Following treatment, improvements were noted in the range of movement of knees and ankles, the perception of pain in both knees, and ankle functionality.

**Conclusions:**

Until now, manual therapy using joint traction was contraindicated in patients with hemophilia and inhibitor, as it was feared to cause possible joint bleeding. This is the first case study to address the safety and efficacy of manual therapy in a patient with hemophilia and an inhibitor. The results of this study may help to establish which manual therapy treatments are indicated in patients with hemophilic arthropathy and inhibitors. Thus, a physiotherapy program based on manual therapy may be safe in patients with hemophilia and inhibitor and such therapy may improve joint condition, pain, and joint range of motion in patients with hemophilia and inhibitor. Randomized clinical trials are needed to confirm the results of this case study.

## Background

Hemophilia is a congenital coagulopathy characterized by the absence of certain clotting factors: factor VIII (Hemophilia A) or factor IX (hemophilia B). Pharmacological treatment involves the intravenous administration of concentrates of the missing clotting factor, either periodically (prophylactic treatment) or in the event of bleeding (on demand) [1].

The development of inhibitors (antibodies) against the exogenous factor administered is the main complication in the treatment of hemophilia. The prevalence of inhibitors is higher in hemophilia A than in hemophilia B. The reported prevalence of inhibitors in unselected haemophilia populations is generally reported to be about 5–7% [2]. These patients present a higher bleeding phenotype, with the added complication of the pharmacological approach needed to control the hemorrhagic process. For this reason, degenerative joint lesions are more common in patients with hemophilia and inhibitors than in those without antibodies against the clotting factor [3].

The deficient production of one of these clotting factors entails, rather than an increase in bleeding, an alteration in the control thereof. Healthy subjects experience bleeding (microhemorrhages) on a daily basis, which their coagulation system controls immediately, so the events go unnoticed. However, in patients with hemophilia these microbleedings are not controlled, triggering muscle and joint bleeding and thus the hemorrhagic clinical pattern characteristic of this disease. In particular, hemophilia is characterized by the development of hemorrhages in the locomotive apparatus, especially in the knee, ankle and elbow joints [4]. Repeated hemorrhagic episodes in the same joint, in the long term lead to the emergence of a progressive joint degeneration known as hemophilic arthropathy [5]. This arthropathy presents, among other symptoms, with chronic pain, decreased joint range of motion and muscle strength, and proprioceptive and gait disorder, when it affects lower limbs [6].

The literature reveals many studies that have assessed the safety and efficacy of different physiotherapy techniques in the approach to the hemophiliac patient [7, 8]. However, one of the most widespread exclusion criteria in most studies on hemophilia is the development of inhibitors. The risk of bleeding, the complexity of hemostatic management and control and the uncertainty in the process of evaluating the safety of a technique, are three of the main reasons for the non-inclusion of patients with inhibitors.

This paper describes a case study to evaluate the safety of a manual physiotherapy intervention in a patient with hemophilia B and inhibitors.

## Case presentation

**Fig. 1:**
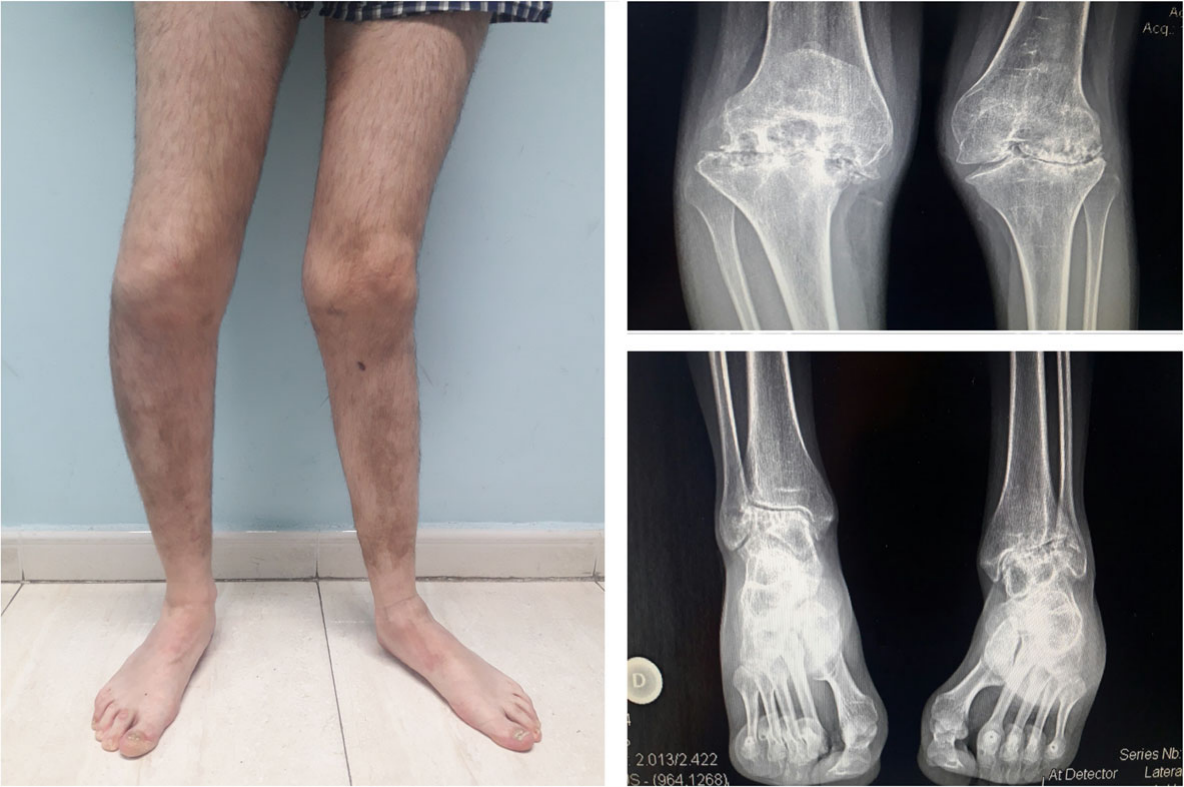
Shows an image of the patient’s lower load-bearing limbs

The patient is 26 years-old, 179 cm in height and weighs 67 kg. At the time of the study the patient was working (active work, eight hours a day), he had a medical diagnosis of bilateral hemophilic arthropathy in knees, ankles and elbows, and was administered daily 60 mg Etoricoxib (selective COX-2 inhibitor) and 150 mg Tramadol (opioid), for pain control. The patient reports to have had moderate-severe pain for more than 3 years, requiring painkillers on a daily basis for 3 years. In the 6 months prior to the study he developed 6 hemarthrosis in the lower limbs (1 in the right knee, 1 in the left knee, and 4 in the left ankle). The patient is on the waiting list to undergo orthopedic surgery for total right knee replacement. In the months prior to the study, he underwent two radiosynovitis interventions (in his right elbow and left ankle joints). His clinical and functional situation is currently affecting his work performance, with a moderate amount of time away from work. The pharmacological treatment received is based on a prophylaxis regime (5 IU of Novoseven, every 48 h). Figure 1 shows the lower limbs and their radiological image (Fig. 1).

**Fig. 2:**
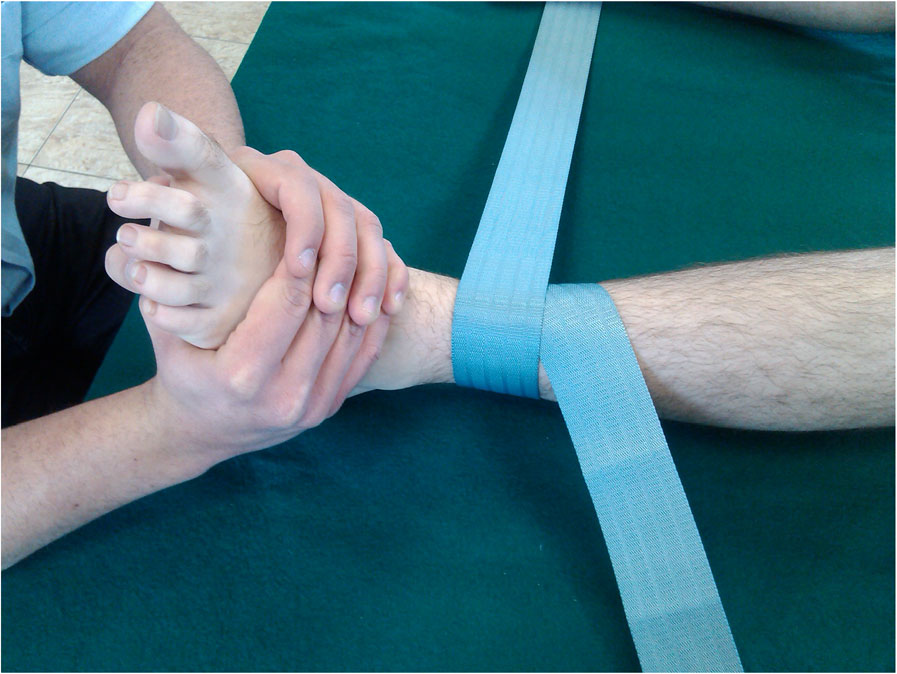
Shows a traction joint of ankle

A manual orthopedic therapy intervention has been carried out by means of range I-II joint traction in knees and ankles [9], for a period of 3 months, with a frequency of two weekly 45-min sessions. In each session, submaximal mobility traction of knees and ankles (80% of the range of motion) was carried out, with the aim of reducing pain and improving knee and ankle mobility. The treatment involved 15 min of joint traction in ankles and another 15 min in knees (grade I–II). The distal tibia and fibula were fixation with straps and the proximal talus was held in place manually with the patient in supine position and the traction was carried out in the submaximal ranges of dorsal and plantar flexion to traction the ankle. Figure 2 shows the joint traction technique of the ankle. For knee joint traction, we placed the patient in the prone position, performing traction (grade I-II) in submaximal flexion and knee extension, using a strap and manually securing the distal part of the femur. Traction was maintained for 15 s, with a 20-s interval between each joint traction [9].

Before and after the intervention three dependent variables were evaluated: range of motion, pain and joint condition. Range of motion of dorsal and plantar ankle flexion and flexion and knee extension were measured using a universal goniometer. The anatomical references used were those identified by Querol [10], using the zero-reference method for the mobile arm of the goniometer, as described by Norkin et al. [11]. Pain perception was measured using the visual analog scale, VAS, with scores ranging from 0 to 10 points (from no pain, to the maximum pain imaginable). Joint condition was evaluated using the Hemophilia Joint Health Score [12]. This scale consists of 8 items per joint (plus Gait being analyzed globally), evaluating *joint swelling, duration of swelling, muscle atrophy, strength, crepitus on motion, flexion* and *extension loss,* and *pain*. The total scores of the updated version of HJHS range from 0 to 124 points (0–20 points for each of the six joints evaluated, plus 4 points for the overall assessment of gait). At baseline, patient was given a self-record of bleeding and a telephonic follow-up was carried out over the 48 h following each treatment session. In this way, intervention safety was assessed, in terms of the occurrence of muscle and joint bleeding in the treatment area.

In addition to the physiotherapy treatment using manual therapy, no further intervention was performed. Moreover, the patient continued with his normal everyday activities throughout the treatment period. Upon completion of the treatment period improvement was noted in almost all ranges of knee and ankle movement. Moreover, improvement was noted in the perception of pain in both knees, and in ankle functionality. However, the main finding of this case study is the absence of hemorrhagic episodes in knees or ankles as a result of the intervention. Table 1 shows the results of dependent variables in both evaluations.

**Table 1:** Main statistical data of the evaluations carried out in the present case study

Variables	Measurement	Assessment		Percentage of improvement
		Baseline	Posttreatment	
Range of motion (degrees)	Flexion right knee (degree)	75	75	0.00
	Flexion left knee	100	117	17.0
	Extension right knee	−18	−17	5.50
	Extension left knee	−16	−6	62.5
	Dorsal flexion right ankle	2	3	50.0
	Dorsal flexion left ankle	−2	−2	0.00
	Plantar flexion right ankle	29	38	62.1
	Plantar flexion left ankle	26	29	11.5
Joint pain (0–10 points)	Right knee	5	4	20.0
	Left knee	3	2	33.3
	Right ankle	0	0	0.00
	Left ankle	2	1	50.0
Joint status (0–20 points)	Right knee	11	11	0.00
	Left knee	10	10	0.00
	Right ankle	10	8	20.0
	Left ankle	9	8	11.1

## Discussion and conclusions

Physiotherapy in general, and manual therapy in particular, are two therapeutic tools recently introduced in the physiotherapy approach to patients with hemophilia, based on scientific evidence. However, there are no studies including patients with inhibitors which would enable verification of the safety of these techniques in this group of patients.

This is the first case study to conduct a physiotherapy intervention using joint traction to assess the safety of manual therapy in hemophiliacs with inhibitors. During the treatment period, the patient with hemophilia and inhibitors suffered no joint or muscle hemorrhages in knees or ankles. Although the patient developed haemarthrosis in the knees, this was caused by trauma, clearly located by the subject at the onset of clinical symptoms. Therefore, it may be concluded that in hemophiliacs with inhibitors, Grade I-II joint traction in knees and ankles appears to be safe, provided it is performed within a range of submaximal mobility and assisted by a manual therapy specialist.

Range of motion improvement noted in knees and ankles is consistent with that observed in other similar studies [9, 13] implementing manual therapy. The results which differ based on the knee or ankle assessed, depend on the clinical condition of the joint. Axial deformities, osteophytosis and the narrowing of the articular space, are three clinical factors that especially limit expectations. Therefore, an approach based on the joints (joint capsule), muscles and fascia, is essential in range of motion improvement.

Chronic pain is one of the most limiting and disabling clinical manifestations in patients with hemophilia [14]. Chronic pain is characteristic of hemophilic arthropathy and has been described as the main cause of disability in these patients, affecting their functional capacity and quality of life [15]. The improvement achieved in the knees, reported by the patient to be the joints with most pain at pretreatment assessment, is substantial. Decompression of the joint space, in addition to elongation of the articular capsule, can relieve pain in patients with hemophilia and inhibitors and significant joint deterioration. To this end, it is vital to develop the technique, strictly following manual therapy criteria and methodology, in order to avoid bleeding episodes.

This study aims to achieve the inclusion of patients with inhibitors in clinical studies using physiotherapy in hemophilia. These patients, who normally suffer more disabling joint symptoms than subjects without inhibitors, need a specialized and evidence-based approach. Establishing well-designed physiotherapy protocols implemented by hemophilia specialists may be the first step in the development of scientific evidence, unavailable to date [16, 17].

Coordination between the various specialists who treat patients with hemophilia (hematologists, nurses, orthopedic surgeons, physiotherapists, etc.) is essential for a proper approach [18].

For patients with inhibitors, whose frequency of bleeding is higher and who reach adulthood in more disabling musculoskeletal conditions, coordination of the interventions among the entire healthcare team is critical. As a step prior to orthopedic surgery, the combined effort of hematologists and physiotherapists is essential to delay surgery as much as possible, maintaining the patient’s functionality and perception of quality of life [19].

## Abbreviations

COX: Cyclooxygenase
